# Altered Autonomic Function in Individuals at Clinical High Risk for Psychosis

**DOI:** 10.3389/fpsyt.2020.580503

**Published:** 2020-11-06

**Authors:** Anna Kocsis, Ruchika Gajwani, Joachim Gross, Andrew I. Gumley, Stephen M. Lawrie, Matthias Schwannauer, Frauke Schultze-Lutter, Tineke Grent-‘t-Jong, Peter J. Uhlhaas

**Affiliations:** ^1^Institute for Neuroscience and Psychology, University of Glasgow, Glasgow, United Kingdom; ^2^Department of Experimental Psychology, Ludwig-Maximilians-Universität München, Munich, Germany; ^3^Institute of Health and Wellbeing, University of Glasgow, Glasgow, United Kingdom; ^4^Department of Psychiatry, University of Edinburgh, Edinburgh, United Kingdom; ^5^Department of Clinical Psychology, University Edinburgh, Edinburgh, United Kingdom; ^6^Department of Psychiatry and Psychotherapy, Medical Faculty, Heinrich-Heine University, Düsseldorf, Germany; ^7^Department of Psychology and Mental Health, Faculty of Psychology, Airlangga University, Surabaya, Indonesia; ^8^Department of Child and Adolescent Psychiatry, Charité Universitätsmedizin, Berlin, Germany

**Keywords:** autonomic functioning, clinical high risk for psychosis (CHR-P), heart-rate variability, resting heart rate, Schizophrenia

## Abstract

**Introduction:** Alterations in autonomic functioning in individuals diagnosed with schizophrenia are well-documented. Yet, it is currently unclear whether these dysfunctions extend into the clinical high-risk state. Thus, we investigated resting heart rate (RHR) and heart rate variability (HRV) indices in individuals at clinical high-risk for psychosis (CHR-P).

**Methods:** We recruited 117 CHR-P participants, 38 participants with affective disorders and substance abuse (CHR-N) as well as a group of 49 healthy controls. CHR-P status was assessed with the Comprehensive Assessment of At-Risk Mental States (CAARMS) and the Schizophrenia Proneness Instrument, Adult Version (SPI-A). We obtained 5 min, eyes-open resting-state MEG data, which was used for the extraction of cardiac field-related inter-beat-interval data and from which heart-rate and heart-rate variability measures were computed.

**Results:** Compared to both CHR-N and healthy controls, CHR-P participants were characterized by an increased RHR, which was not explained by differences in psychopathological comorbidity and medication status. Increased RHR correlated with the presence of subthreshold psychotic symptoms and associated distress. No differences between groups were found for heart-rate variability measures, however. Furthermore, there was an association between motor-performance and psychophysiological measures.

**Conclusion:** The current study provides evidence of alterations in autonomic functioning as disclosed by increased RHR in CHR-P participants. Future studies are needed to further evaluate this characteristic feature of CHR-P individuals and its potential predictive value for psychosis development.

## Introduction

Schizophrenia (SZ), the most severe manifestation of psychosis with a lifetime prevalence of ~1% (1), is accompanied by a significantly decreased life expectancy (2, 3). Recent evidence suggests cardiovascular diseases (CVD) may contribute significantly toward increased mortality in SZ (4). Furthermore, the prodromal phase of SZ has been associated with a substantially higher risk of physical conditions, such as hypertension, heart disease, and cerebrovascular disease (5). In addition to SZ, altered autonomic functioning has been associated with a range of psychiatric syndromes, including anxiety, depression and personality disorders (6–9).

Resting heart-rate (RHR) is a non-invasive measure of vagal tonic inhibitory control over autonomic function (10, 11) and is determined by intrinsic cardiac mechanisms as well as the activity of both branches of the autonomic nervous system (ANS)—the sympathetic and the parasympathetic (vagus) nerves. At rest, the vagal tone reduces RHR to 60–80 beats per minute (12). A heart rate (HR) higher than the RHR is a result of both the withdrawal of vagal tone and the activation of the sympathetic branch of the ANS (11, 13).

Animal and human data have suggested that cortical activity, especially in prefrontal areas, plays a modulatory role in cardiovascular function (10). A potential pathway for the modulatory role of frontal areas is through the tonic inhibition of the amygdala (14, 15). Specifically, inhibition increases the dominance of vagal influence on cardiovascular activity during rest.

There is emerging evidence that increased RHR may be associated with SZ. For example, a study by Latvala et al. (16) reported that elevated RHR is associated with increased risk for developing SZ. In addition, a number of studies have reported increased RHR in unmedicated first episode SZ patients (17–22). Medication status is important in light of substantial evidence found for effects of atypical antipsychotics on RHR and HR variability (HRV), although this relationship may not hold for other psychiatric disorders (23). Furthermore, psychotic symptom severity has been found to correlate with cardiac ANS disturbances (17). Reinertsen et al. (24) reported that cardio-vascular activity (HR) was associated with illness severity in SZ.

To date, few studies have investigated HR changes in participants that meet criteria for clinical high-risk of psychosis (CHR-P). CHR-P criteria include ultra-high-risk (UHR) criteria (i.e., based on attenuated or transient psychotic symptoms, in addition to genetic risk plus functional deterioration) (25), as well as basic symptoms (BS)—self-experienced perceptual and cognitive disturbances (26, 27). Clamor et al. (28) found a significant increase in RHR in participants with established psychotic disorders, but not in participants meeting UHR criteria. In contrast, Counotte et al. (29) reported associations between psychosis liability (based on UHR criteria and genetic risk for psychosis) and increased HR as well as decreased HRV during a virtual reality experiment with social stressors.

In the present study, we aimed to further investigate altered autonomic functioning in a population of CHR-P individuals (*n* = 117) to assess whether aberrant HR may be present in the psychosis-risk state. To examine the diagnostic specificity of alterations in ANS, we also assessed identically recruited participants who did not met CHR-P criteria but were characterized by substance abuse and affective disorders (CHR-N = 38) as well as a group of healthy controls (CON = 49).

The psychophysiological measures of cardio-vascular activity were extracted from a 5-min resting-state magnetoencephalography (MEG) recording. The contraction of the heart-muscle generates a strong electrical dipole resulting in a magnetic field [the cardiac field artifact, CFA (30)], which is visible in the PQRST cycle of the electrocardiogram. The CFA signal propagates throughout the body and can be recorded by electrodes positioned at any location. Independent component analysis on MEG data was used to visually identify and extract the heart-rate signal, that is, the inter-beat-interval (IBI) data. In line with recent reports of autonomic changes in the CHR-P individuals (31) and hypothalamic-pituitary-adrenal axis disturbances in first episode psychosis (FEP) patients (32), we expected a significant increase in RHR, as well as a decrease in HRV indices in the CHR-P sample but not in the CHR-N group.

## Materials and Methods

### Participants

A total of 204 participants were recruited as part of the “Youth Mental Health Risk and Resilience Study” [YouR-Study; (32)], a Medical Research Council funded study aiming at identifying biological and psychological markers of psychosis-risk. Participants were recruited through an online-screening approach [for more detail on the recruitment procedure, see (33)]. The Comprehensive Assessment of At-Risk Mental States (CAARMS) Interview (25) as well as the Schizophrenia Proneness Instrument (SPI-A) (26) were used for establishing CHR-P criteria. For the SPI-A, Cognitive-Perceptive Basic Symptoms (COPER) and Cognitive Disturbances (COGDIS) items were administered. CAARMS-criteria were established as follows: (a) criteria for the attenuated psychosis group (attenuated psychotic symptoms present in the last year without a decline in functioning); (b) criteria for genetic risk with functional decline (30% drop in global functioning score); and (c) criteria for the brief limited intermittent psychotic symptoms (BLIPS) group. Participants who did not meet a CHR-P criterion, were assigned to the CHR-N group (*n* = 38). CHR-N participants were recruited through the same pathway as CHR-P participants but did not meet BS/UHR-criteria. The rationale was to have a comparison group that is more closely matched for co-morbidity frequently associated with CHR-P participants, such as affective disorders, substance abuse and lower functioning. Finally, a group of 49 healthy participants (CON) were recruited without an Axis I diagnosis or family history of psychotic disorders. All participants were between 16 and 35 years of age and were excluded for current or past diagnosis with Axis I psychotic disorders [see (32) for more details].

The M.I.N.I. International Neuropsychiatric Interview [M.I.N.I. 6.0; (16)], the scales for premorbid adjustment (34), and social and role functioning (35) were administered. Neuropsychological assessment consisted of the Brief Assessment of Cognition in Schizophrenia [BACS (36)], as well as three tasks from the Penn Computerized Neurocognitive Battery [CNB (37); the Continuous Performance Test, the N-Back Task, and the Emotion Recognition Task].

### Psychophysiological Data Acquisition and Analysis

Five-minute, eyes-open resting-state MEG data was acquired using a 248-channel 4D-BTI magnetometer system (MAGNES 3600 WH, 4D-Neuroimaging, San Diego), at a sampling frequency of 1,017.25 Hz, filtered online between DC and 400 Hz. Four minutes of MEG resting-state data were extracted, down sampled to 400 Hz and bandpass-filtered between 5 and 70 Hz.

A continuous single epoch data was created for each channel using the open-source MATLAB Fieldtrip Toolbox (38). The data were then submitted to Independent Component Analyses (ICA) to isolate the component best describing the R-peaks of the heartbeat signal. The resulting single vector of time-varying heartbeat signal was further analyzed using the validated HRV-analyses Matlab Toolbox ARTiiFACT (39).

Firstly, time-series data were low-pass filtered at a manually adjustable cut-off frequency, and R-peaks were detected using a global threshold detection method that was manually adjusted between 0 and 20 μV for each participant. Secondly, visual inspection of IBIs was performed in order to detect extra or missing IBIs and manually correct them. Thirdly, automatically detected artifacts were corrected using cubic spline interpolation.

Four parameters were extracted: (1) RHR, (2) the square root of the mean squared differences of successive normal-to-normal intervals (RMSSD), and (3) the standard deviation of normal-to-normal intervals (SDNN). Fast Fourier transformation was computed with frequency bands set as high frequency (HF, 0.15–0.4 Hz), low frequency (LF, 0.04–0.15 Hz), and very low frequency (VLF, <0.04 Hz). Finally, 4) the ratio of LF and HF (LF/HF), reflecting sympatho-vagal balance (40), was computed.

### Statistical Analysis

All statistical analyses were performed using the *R* statistical software (41). Demographic and clinical parameters were assessed using Welch based *F*-tests or Kruskal–Wallis *H*-tests. Independent-sample Kruskal–Wallis *H*-tests were used to investigate main effects (i.e., relationship between psychophysiological measures and clinical category (CON – CHR-N – CHR-P; with the psychophysiological measure as the outcome and the clinical category as the predictor: outcome ~ predictor). Eta-squared (η^2^) with bias-corrected and accelerated (BCa) 1,000 samples bootstrapped confidence interval (95% CI) is reported as an indicator of effect size. For significant group effects, Hochberg-corrected, 20% means-trimmed, and 3,000 samples bootstrapped *post-hoc* comparisons were calculated.

Covariates were included to investigate possible effects including smoking (i.e., number of cigarettes smoked per day, current or past), BMI (42), age, and sex (43), medication (7, 23) (antipsychotics, antidepressants, beta-blockers, anxiolytics, stimulants, mood stabilizers, and combinations of these were considered; for details (see Supplementary Table 1)]. We conducted a parametric ANCOVA with clinical status (CON – CHR-N – CHR-P) as the first and the covariates as remaining predictors. Orthogonal Helmert contrasts were used. The assumption of the homogeneity of regression slopes was examined by including the interaction terms in the model. Tukey *post-hoc* tests on adjusted means were carried out. Partial omega squared (ω^2^_*p*_) was reported as a measure of effect size. To further investigate the influence of covariates, we conducted within-group analysis for the CHR-N and CHR-P groups separately. When assumptions for within-group analysis of variance were met, we conducted parametric ANOVAs with η^2^ as effect size.

To investigate the relationship between psychophysiological measures and clinical symptomatology and presentation, CAARMS severity, CAARMS distress, and the comorbidity index were computed for each participant and entered into linear regression and correlational analyses. Spearman's two-sided correlation with BCa 2,000 samples bootstrap 0.95 CI, corrected for ties (ρ [95% CI], *p*), are reported.

A factor analysis was conducted to assess the effect of psychopathological comorbidity expressed through the comorbidity index variable that was obtained from the M.I.N.I. ratings. In the first step, all M.I.N.I. variables were used except “Antisocial personality disorder,” “Anorexia,” and “Anorexia Binge,” due to missing data. Additional variables were excluded from the factor analysis due to low *r*-drop values (“Panic No Agoraphobia,” *r*-drop = 0.14; “Agoraphobia,” *r*-drop = 0.13), resulting in the final test reliability expressed in standardized alpha of 0.83 (0.78, 0.85). The appropriateness of conducting a factor analysis on these data was confirmed via the Bartlett test (chi-square = 1,727.90, *p* < 0.001 ^***^, *df* = 276, det < 0.001^***^). Several factor analysis models were tested by performing Pearson's correlation and replacing missing values with medians [see (31)]. The best performing model was the maximum likelihood estimation (BIC = −252.07). Factor loadings were extracted for each participant (the “Comorbidity index” variable).

BACS and CBN Emotion Recognition raw scores were z-transformed to the CON and corrected for sex (44). Non-parametric Spearman's rho correlations were calculated for psychophysiological measures and both BACS and CBN Emotion Recognition data, separately for each group. As these analyses were explorative, no corrections for multiple comparisons were implemented.

## Results

Sample characteristics are presented in Table 1. The groups differed significantly with respect to years of education and psychopathological comorbidity, with CHR-N participants having significantly lower years of education and higher comorbidity scores. Furthermore, both the CHR-P and CHR-N groups had significantly higher medication scores than CON. Additionally, the groups were significantly different in terms of CAARMS severity and distress. For a detailed presentation of clinical characteristics (see Supplementary Table 2).

**Table 1:** Sample characteristics.

	**CON**	**CHR-N**	**CHR-P**	**GROUP effect**			**Pairwise comparisons**
	**(*n* = 49)**	**(*n* = 38)**	**(*n* = 117)**	***F*, *p***	**χ^**2**^, *p***	**η^**2**^**	**psihat, *p* (*p* crit)**
**Age**, years (SE)	23 (0.51)	23 (0.75)	22 (0.41)	0.90, 0.410		0.01	
**Sex**, female/male (%female)	33/16 (67.35)	27/11 (69.23)	87/30 (74.36)	0.47, 0.627		0.00	
**Education**, years (SE)	16.61 (0.42)	16.49 (0.56)	15.21 (0.31)	4.42, 0.015*		0.04	CHR-N vs. CON: ns CHR-N vs. CHR-P: 1.01, 0.077 (0.025) CON vs. CHR-P: 1.43, **0.003 (0.017)** **
**Smoking**, mean (SE)	1.00 (0.32)	1.22 (0.36)	1.85 (0.26)		3.22, 0.200	0.01	
**BMI**, mean (SE)	3.92 (0.07)	4.16 (0.11)	4.12 (0.10)		3.75, 0.154	0.01	
**Comorbidity Factor** mean (SE)	−0.81 (0.01)	−0.33 (0.10)	0.45 (0.10)		101.18, < 0.001***	0.49	CHR-N vs. CON: 0.35, < 0.001*** CHR-N vs. CHR-P: −0.76, < 0.001*** CON vs. CHR-P: −1.11, < 0.001***
**Medication** *n* (medicated)	1	14	55		30.285, < 0.001***	0.14	CHR-N vs. CON: 0.56, 0.012 (0.025)** CHR-N vs. CHR-P: ns CON vs. CHR-P: −0.95, < 0.001***
**CAARMS severity**, mean (SE)	-	6.21 (0.98)	28.63 (1.55)	162.26, < 0.001***		0.50	CHR-N vs. CON: 5.12, < 0.001*** CHR-N vs. CHR-P: −22.65, < 0.001*** CON vs. CHR-P: −27.77, < 0.001***
**CAARMS distress**, mean (SE)	-	34.33 (7.31)	120.86 (8.19)	92.59, < 0.001***		0.36	CHR-N vs. CON: 21.88, < 0.001*** CHR-N vs. CHR-P: −89.69, < 0.001*** CON vs. CHR-P: −111.57, < 0.001***

*Welch's ANOVA for unequal variance (F, alpha = 0.05, 2-sided) or Kruskal–Wallis H-tests (χ^2^, alpha = 0.05), with eta-square as indicator of effect size are reported. Pairwise comparisons are Hochberg-corrected, 20% means-trimmed, 3,000 samples bootstrapped [psihat/p (p crit)]. CON, healthy controls; CHR-N, clinical high risk-negative; CHR-P, clinical high risk-positive; SE, standard error of mean*.

**Figure 1:**
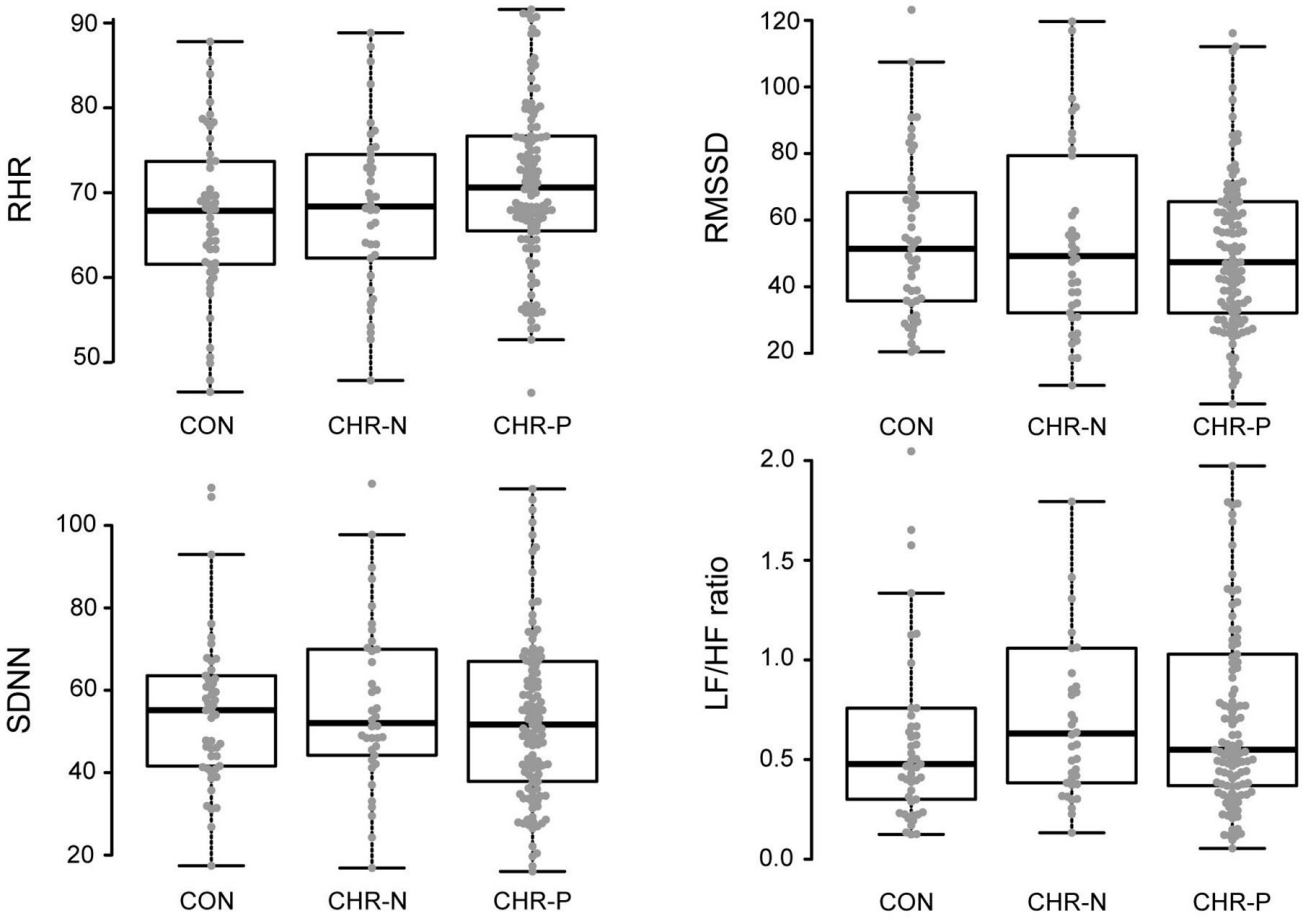
Analyses of variance (mean levels and standard error) and *post-hoc* group comparisons. CON, healthy controls; CHR-N, clinical high risk-negative; CHR-P, clinical high risk-positive; RHR, resting heart rate; RMSSD, square root of the mean squared differences of successive normal-to-normal intervals; SDNN, standard deviation of normal-to-normal heart beat intervals; LF/HF, ratio of low and high frequency power.

Psychophysiological measures indicated higher RHR for the CHR-P group (mean/SE: 71.63/1.03) compared to CON (67.16/1.37), but not to the CHR-N group (68.31/1.59). This was confirmed by Kruskal–Wallis *H* tests, revealing marginally significant group differences for RHR (χ2 = 5.98, *p* = 0.050, η^2^ = 0.19 [−0.01, 0.08]). Pairwise comparisons showed the expected significant increase in RHR only in the CHR-P group relative to CON (psihat = −3.97, *p* = 0.015, *p*_crit_ = 0.016; see Figure 1). In contrast, no significant group effects were found in HRV indices (RMSSD/SDNN) or changes in sympathovagal balance (LH/HF) (Table 2).

**Table 2:** Estimated means (standard error) and analysis of group differences for psychophysiological measurements.

	**CON**	**CHR-N**	**CHR-P**	**GROUP effect**		**Pairwise comparisons**
	**(*n* = 49)**	**(*n* = 38)**	**(*n* = 117)**	**χ^**2**^, *p***	**η^**2**^ (95% CI)**	**psihat/*p* (*p* crit)**
**Resting heart rate (RHR)**, mean (SE)	67.16 (1.37)	68.31 (1.59)	71.63 (1.03)	5.98, 0.050*	0.19 [−0.01, 0.08]	CHR-N vs. CON: ns CHR-N vs. CHR-P: ns CON vs. CHR-P: −3.97/ 0.015 (0.016)*
**Time-domain HRV**						
**RMSSD**, mean (SE)	56.08 (4.02)	55.53 (5.16)	53.03 (2.79)	0.61, 0.738	−0.01 [−0.01, 0.00]	
**SDNN**, mean (SE)	57.01 (3.13)	56.40 (3.28)	54.91 (2.17)	0.78, 0.677	−0.01 [−0.01, 0.01]	
**Frequency-domain HRV**						
**LF/HF**, mean (SE)	0.75 (0.11)	1.03 (0.22)	0.93 (0.11)	2.54, 0.281	0.00 [−0.01, 0.04]	

*Kruskal–Wallis H-tests (χ^2^, alpha = 0.05) are reported. Eta-squared (η^2^) with bias-corrected and accelerated (BCa) 1,000 samples bootstrapped confidence interval [95% CI] is reported as an indicator of effect size. Pairwise comparisons are Hochberg-corrected, 20% means-trimmed, 3,000 samples bootstrapped [psihat/p (p crit)]. CON, healthy controls; CHR-N, clinical high risk-negative; CHR-P, clinical high risk-positive; RHR, resting heart rate; RMSSD, square root of the mean squared differences of successive normal-to-normal intervals; SDNN, standard deviation of normal-to-normal heart beat intervals; LF/HF, ratio of low and high frequency power; SE, standard error of mean*.

A parametric ANCOVA that included age, sex, smoking, and BMI as covariates revealed a significant between-group effect for RHR [*F*_(2, 168)_ = 3.93, *p* = 0.021, ω^2^_*p*_ = 0.03] as well as a significant effect of sex [*F*_(1, 168)_ = 7.48, *p* = 0.007, ω^2^_*p*_ = 0.02] and age [*F*_(1, 168)_ = 3.91, *p* = 0.05, ω^2^_*p*_ = 0.02]. Tukey *post-hoc* tests on adjusted means revealed a significant CON vs. CHR-P difference (Δ = 5.12, *t* = 2.64, *p* = 0.024).

Within-group analysis of the relationship between psychophysiological measures and medication or comorbidity status revealed that both medication and comorbidity factor had a significant effect only on the LF/HF measurement and only in the CHR-P group (see Table 3). No effects were observed for RHR.

**Table 3:** Within-group effects of medication and comorbidity factor on psychophysiological measurements.

	**Medication**				**Comorbidity Factor**			
	**CHR-N**		**CHR-P**		**CHR-N**		**CHR-P**	
	***F*, *p***	**η^**2**^**	***F*, *p***	**η^**2**^**	***F*, *p***	**η^**2**^**	***F*, *p***	**η^**2**^**
**MEANHR**	1.65, 0.207	0.01	0.84, 0.361	0.06	2.06, 0.160	0.11	0.03, 0.865	0.00
**RMSSD**	0.18, 0.67	0.03	0.51, 0.474	0.08	1.84, 0.184	0.21	0.91, 0.343	0.01
**LF/HF**	0.30, 0.590	0.06	9.96, 0.002*	−0.02	0.54, 0.466	−0.14	3.10, **0.081**	0.03
**SDNN**	0.84, 0.366	0.05	0.89, 0.349	0.08	0.62, 0.437	0.32	0.76, 0.386	0.01

*A parametric ANOVA (F, alpha = 0.05). CHR-N, clinical high risk-negative; CHR-P, clinical high risk-positive; RHR, resting heart rate; RMSSD, square root of the mean squared differences of successive normal-to-normal intervals; SDNN, standard deviation of normal-to-normal heart beat intervals; LF/HF, ratio of low and high frequency power*.

**Figure 2:**
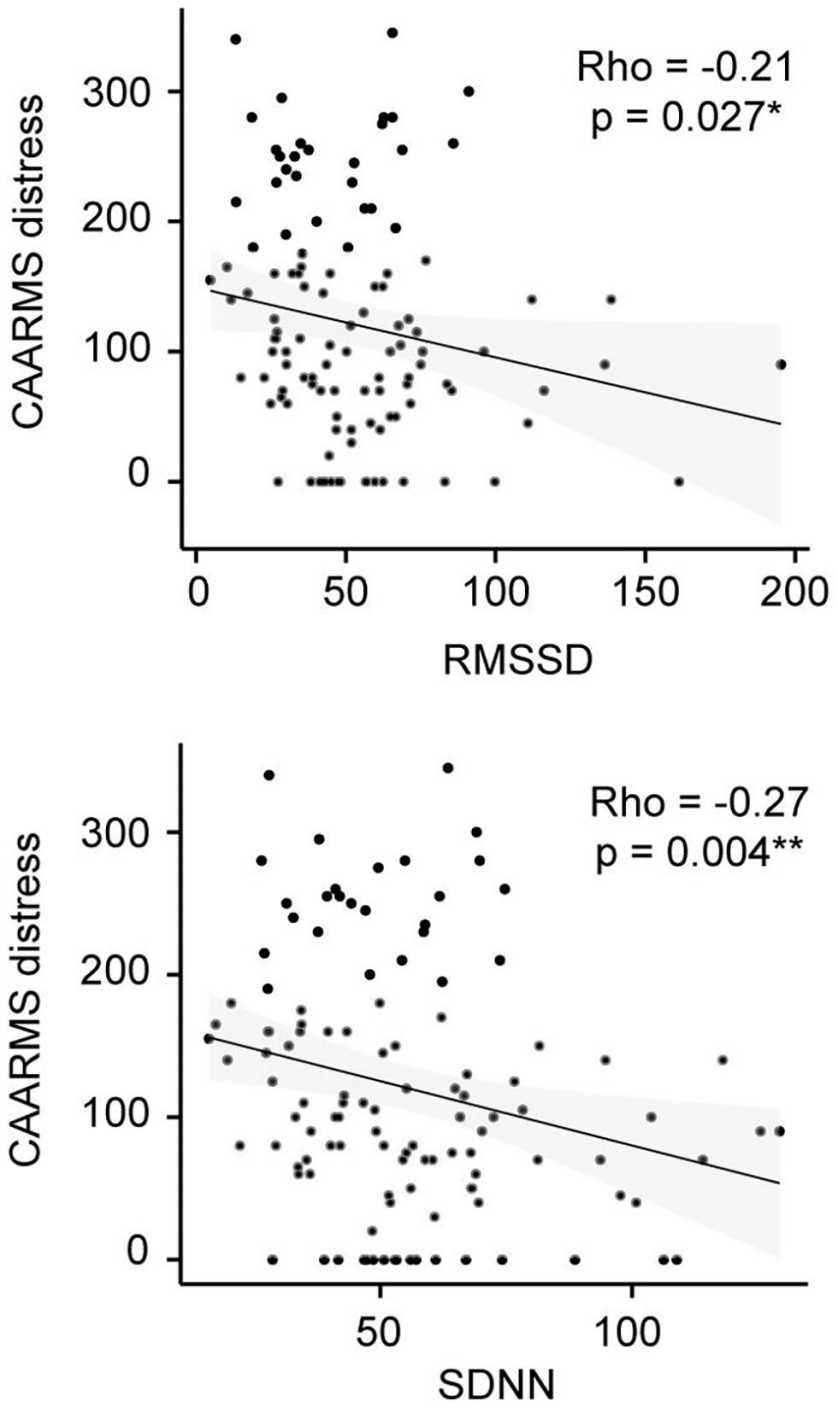
CAARMS Distress correlation analysis in the CHR-P group. Non-parametric Spearman's correlation is represented. RMSSD, square root of the mean squared differences of successive normal-to-normal intervals; SDNN, standard deviation of normal-to-normal heart beat intervals.

In addition, correlation analyses revealed no significant relationships between the comorbidity factor and any of our psychophysiological measures (Table 4). Yet, there were significant correlations between CAARMS distress and both SDNN (ρ = −0.27, *p* = 0.004) and RMSSD (ρ = −0.21, *p* = 0.027) in the CHR-P group only (Figure 2). However, these did not survive correction for multiple comparisons. Linear regression analyses showed a trend-level relationship between RHR and CAARMS severity [adjusted *R*^2^ = 0.013, *F*_(1, 199)_ = 3.764, *p* = 0.053] as well as distress [adjusted *R*^2^ = 0.014, *F*_(1, 201)_ = 3.887, *p* = 0.050]. In addition, CAARMS distress but not severity was associated with RMSSD [adjusted *R*^2^ = 0.014, *F*_(1, 201)_ = 4.014, *p* = 0.046] and SDNN [adjusted *R*^2^ = 0.023, *F*_(1, 201)_ = 5.692, *p* = 0.018]. There were no significant correlations between BS severity or distress with any of the psychophysiological measures (see Supplementary Table 3).

**Table 4:** Correlations of psychophysiological measurements with CAARMS severity and distress, and the comorbidity factor.

	**CAARMS severity**			**CAARMS distress**			**Comorbidity factor**		
	**CON**	**CHR-N**	**CHR-P**	**CON**	**CHR-N**	**CHR-P**	**CON**	**CHR-N**	**CHR-P**
**RHR**	-	−0.11 [−0.47, 0.23] 0.503	0.01 [−0.19, 0.20] 0.921	-	−0.08 [−0.41, 0.23] 0.619	0.07 [−0.12, 0.25] 0.477	-	−0.21 [−0.50, 0.18] 0.214	0.00 [−0.19, 0.18] 0.974
**RMSSD**	-	0.05 [−0.28, 0.34] 0.783	−0.07 [−0.26, 0.11] 0.434	-	−0.03 [−0.34, 0.29] 0.847	−0.21 [−0.37, −0.02] 0.027*	-	0.13 [−0.23, 0.47] 0.421	−0.074 [−0.26, 0.11] 0.427
**SDNN**	-	0.07 [−0.27, 0.38] 0.675	−0.11 [−0.28, 0.06] 0.229	-	−0.02 [−0.34, 0.31] 0.887	−0.27 [−0.42, −0.10] 0.004**	-	0.11 [−0.27, 0.45] 0.513	−0.05 [−0.23, 0.13] 0.561
**LF/HF**	-	−0.02 [−0.31, 0.26] 0.919	−0.07 [−0.26, 0.12] 0.458	-	0.11 [−0.19, 0.40] 0.495	0.01 [−0.16, 0.18] 0.878		−0.15 [−0.44, 0.19] 0.376	−0.03 [−0.24, 0.16] 0.752

*Spearmans's two-sided correlation with bias-corrected and accelerated (BCa) 2,000 samples bootstrap 0.95 confidence interval, corrected for ties (ρ [95% CI], p). CON, healthy controls; CHR-N, clinical high risk-negative; CHR-P, clinical high risk-positive; HRV, heart rate variability; SDNN, standard deviation of normal-to-normal heart beat intervals; RMSSD, square root of the mean squared differences of successive normal-to-normal intervals; HF, high frequency power*.

**Figure 3:**
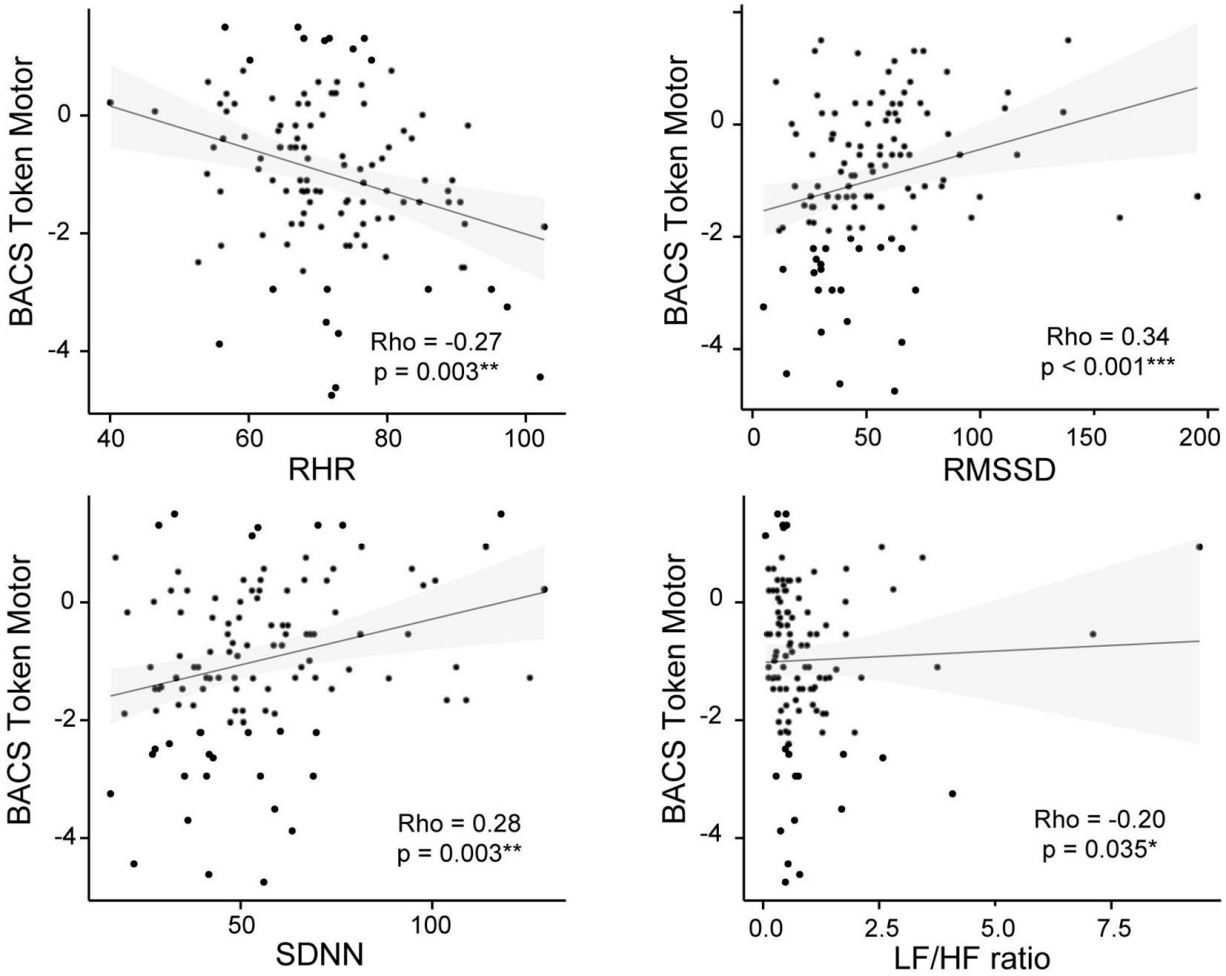
BACS Token Motor and psychophysiological measures correlation plots for the CHR-P group. Non-parametric Spearman's correlation is represented. RHR, resting heart rate; RMSSD, square root of the mean squared differences of successive normal-to-normal intervals; SDNN, standard deviation of normal-to-normal heart-beat intervals; LF/HF, ratio of low and high frequency power.

Correlation analysis revealed that the token-motor component of the BACS showed significant correlation with all psychophysiological measures, in particular with RMSSD (ρ = 0.45, *p* = < 0.001; see Figure 3). There were no significant results for the remaining neurocognitive measures (Table 5).

**Table 5:** Correlations of psychophysiological measurements with cognitive and emotion recognition tests.

	**RHR**			**RMSSD**			**SDNN**			**LF/HF**		
	**CON**	**CHR-N**	**CHR-P**	**CON**	**CHR-N**	**CHR-P**	**CON**	**CHR-N**	**CHR-P**	**CON**	**CHR-N**	**CHR-P**
**BACS**												
Token motor	ns	ns	−0.27 [−0.46, −0.10] 0.003**	ns	ns	0.34 [0.15, 0.51] < 0.001***	ns	ns	0.28 [0.08, 0.44] 0.003**	ns	ns	−0.20 [−0.36, −0.02] 0.035*
Symbol coding	−0.3 [−0.53, 0.00] 0.039*	ns	−0.16 [−0.34, 0.03] **0.089**	ns	ns	ns	ns	ns	ns	ns	−0.27 [−0.56, 0.08] **0.098**	−0.16 [−0.34, 0.03] **0.092**
**CNB—Emotion recognition task**												
Correct anger	ns	ns	ns	ns	ns	ns	ns	ns	−0.18 [−0.34, −0.01] **0.052**	ns	ns	ns
Correct fear	ns	ns	ns	−0.24 [−0.51, 0.07] **0.093**	ns	ns	−0.27 [−0.53, 0.03] **0.061**	ns	ns	ns	ns	ns
Correct happy	−0.33 [−0.57, 0.02] 0.022*	ns	ns	ns	ns	ns	ns	ns	ns	0.29 [−0.01, 0.51] **0.048**	ns	0.16 [−0.04, 0.32] **0.090**
Correct no emotion	ns	ns	ns	ns	ns	ns	ns	ns	ns	ns	ns	−0.19 [−0.35, −0.01] 0.047*
Correct sad	ns	ns	ns	ns	ns	ns	ns	−0.35 [−0.62, 0.02] 0.033*	ns	ns	ns	ns

*Spearmans's two-sided correlation with bias-corrected and accelerated (BCa) 2,000 samples bootstrap 0.95 confidence interval, corrected for ties (ρ [95% CI], p). BACS and CBN scores were standardized to control group data, controlled for sex category. A Composite score was calculated following (44). CON, healthy controls; CHR-N, clinical high risk-negative; CHR-P, clinical high risk-positive; RHr, resting heart rate; RMSSD, square root of the mean squared differences of successive normal-to-normal intervals; SDNN, standard deviation of normal-to-normal heart beat intervals; LF/HF, ratio of low and high frequency power*.

## Discussion

The current study investigated RHR abnormalities in a sample of CHR-P participants. Cardiovascular activity indices were computed by analyzing the time-varying heartbeat signal obtained directly from resting-state MEG data, which contains the ballisto-cardiogram artifact that is likely caused by blood-flow in the vessels around the head/neck area (45). These indices are thus comparable to the values reported in previous studies using finger photoplethysmography (28, 29), a technique which measures HR indices from blood volume pulses. We found a significant increase in RHR in the CHR-P group compared to healthy controls but not to the CHR-N group. Importantly, the observed difference in RHR was not influenced by several covariates, including age, sex, smoking habits, medication, and BMI.

Previous studies that have investigated cardiovascular changes in smaller psychosis-risk samples have reported conflicting results. Clamor et al. (28) did not find an increase in RHR or a reduction in HRV in CHR-P participants. Interestingly, RHRs for the CHR-P group were similar to those found in our study. However, the HC group in the current study was characterized by lower RHR compared to Clamor et al. (28). Counotte et al. (29) reported both increased HR and reduced HRV in both 22 UHR and 44 first-degree relatives of patients with psychosis. It is important to note, however, that HR was measured during a virtual-reality experiment involving social stressors.

Interestingly, the Counotte study found no changes in recorded levels of skin conductance, a pure measure of sympathetic nervous-system (SNS) activation. This is in line with evidence suggesting that dysfunctional parasympathetic nervous-system (PNS) activity in SZ may be considered as the main contributor to sympathovagal imbalance (9, 46, 47). In the current study, we only found evidence for an increase in RHR, but not in HRV measures such as RMSSD, SDNN, or LF/HF in CHR-P participants. RHR is regarded as a tonic and much more stable measure of ANS functioning than HRV measures, which are highly influenced by breathing rhythm [for example see (48)]. RHR has been associated with severity of psychotic symptoms in chronic SZ patients (49) specifically in terms of changes in PNS functioning, evident in the cardiac vagal index. Specifically, baseline RHR predicted the changes in clinical state on subsequent assessment whereby SZ-patients with elevated RHR showed an increase in the severity of psychosis. The relationship between ANS dysregulation and severity of psychosis was also supported by our observation of a positive relationship between RHR and CAARMS severity and distress.

It has been suggested that ANS dysfunction in SZ is coupled with decreased resting PNS activity, mediated through impaired vagal control in the absence of changes in SNS activity [e.g., review by (46)]. However, the underlying mechanisms for this autonomic imbalance are still unclear. An increase in arousal would be the simplest explanation for increased RHR, but this is unlikely to occur without changes in SNS activity.

Recent findings suggest the vagal nerve has an important influence on immune functioning in SZ patients (50). According to recent meta-analyses, the low-grade inflammatory profile found in SZ patients is correlated with clinical symptom severity (51–53). Furthermore, this inflammatory profile has been reported in drug naïve FEP patients (53). Finally, increased CVD risk in SZ—typically linked to traditional risk factors such as lifestyle, or metabolic syndrome—has recently been shown to bear a relation to systemic inflammation, indicated by increases in leucocyte counts and cytokines concentration (54). Vagal nerve stimulation has been shown to have anti-inflammatory properties [see review (55)] and is potentially involved in inflammation in SZ (50).

A limitation of the present study is that we did not meet the recommended minimum of 5 min for the purposes of cross-study comparison (56), nor did we measure breathing rates. Some studies have shown increased RHRs in conjunction with increased breathing rates in SZ patients [e.g., (18)]. Finally, we also did not assess comorbidities in terms of potential physical illnesses that could alter RHR.

In conclusion, the current data suggest that increased RHR is associated with individuals meeting CHR-P criteria and correlates with severity and distress of increased APS. Given the importance of CVD in SZ (4), it is important to determine the underlying factors that give rise to alterations in RHR in CHR-P individuals. Given recent suggestions of a potential link with neuroinflammation (57), future studies should also further investigate the link between the CHR-P state, aberrant RHR activity, and neuroinflammatory markers. In addition, follow-up data are needed to evaluate its potential as a biomarker for the development of a psychotic disorder.

## Data Availability Statement

The raw data supporting the conclusions of this article will be made available by the authors, without undue reservation.

## Ethics Statement

The study was approved by the ethical committees of University of Glasgow and the National Health Services Research Ethical Committee Glasgow and Greater Clyde. All participants provided written informed consent.

## Author Contributions

AK preformed the data analysis. AK, PU, and TG-'t-J wrote the manuscript. PU conceptualized the project, oversaw design, interpretation, analyses, and edited the manuscript. RG, JG, AG, SL, MS, and FS-L edited the manuscript. All authors contributed to the article and approved the submitted version.

## Conflict of Interest

The authors declare that the research was conducted in the absence of any commercial or financial relationships that could be construed as a potential conflict of interest.
