# Supplementary material for: Altered Autonomic Function in Individuals at Clinical High Risk for Psychosis
Source: Front Psychiatry. 2020 Nov 6;11:580503. doi: 10.3389/fpsyt.2020.580503 (PMC7677235; doi:10.3389/fpsyt.2020.580503)
Supplement: Supplementary file 1 [file Data_Sheet_1.docx]

**Altered autonomic function in individuals at clinical high risk for psychosis**

**Anna Kocsis MSc^1,2^, Ruchika Gajwani, Ph.D.^3^ , Joachim Gross, Ph.D.^1^, Andrew I. Gumley, Ph.D.^3^, Stephen M. Lawrie M.D.^4^, Matthias Schwannauer, Ph.D.^5^, Frauke Schultze-Lutter^7^, Ph.D., Grent-‘t-Jong, Tineke Ph.D.^1,8*^ & Peter J. Uhlhaas, Ph.D.^1,8±*^**

^1^Institute for Neuroscience and Psychology, Univ. of Glasgow, Glasgow, UK

^2^Department of Experimental Psychology, Ludwig-Maximilians-Universität München, Munich, Germany

^3^Institute of Health and Wellbeing, Univ. of Glasgow, Glasgow, UK

^4^Department of Psychiatry, Univ. of Edinburgh, Edinburgh, UK

^5^Department of Clinical Psychology, Univ. Edinburgh, Edinburgh, UK

^7^Department of Psychiatry and Psychotherapy, Medical Faculty, Heinrich Heine University,

Düsseldorf, Germany

^8^Department of Child and Adolescent Psychiatry, Charité Universitätsmedizin, Berlin, Germany

* shared senior authorship

**Correspondence:**

Dr. Peter J. Uhlhaas

Email: peter.uhlhaas@charit.de

Tel: 0049/516193

**Keywords:** Autonomic functioning; Clinical high risk for psychosis (CHR-P); Heart-rate variability; Resting heart rate.

Abstract: 235 words

Main text: 2780 words

Figures: 5

Tables: 3

Appendix A: Supplementary material

**Supplementary Table 1.** Medication, smoking, and Body-mass index (BMI) variables.

|  | **CON**  (n = 49) | **CHR-N**  (n = 38) | **CHR-P**  (n = 117) |
| --- | --- | --- | --- |
| **Medication**, *n* |  |  |  |
| None | 48 | 25 | 60 |
| Anti-psychotic(s) (AP) | - | - | 1 |
| Anti-depressant(s) (AD) | - | 9 | 28 |
| Beta-blocker (BB) | 1 | 2 | 7 |
| AP + AD | - | - | 1 |
| AP + AD + BB | - | - | - |
| AP + AD + anxiolytic (including sedative or mood stabilizer) | - | - | 1 |
| AD + BB | - | - | 4 |
| AD + BB + anxiolytic (including sedative or mood stabilizer) | - | - | 1 |
| AD + anxiolytic (including opioid and sedative) | - | - | 4 |
| AD + stimulant/allergies | - | 1 | 2 |
| AD + mood stabilizer | - | - | 2 |
| Stimulant/allergies | - | 2 | 2 |
| Anxiolytic | - | - | 2 |
| **Smoking**, *n (number of cigarettes smoked in a day)* |  |  |  |
| No present or past | 36 | 24 | 71 |
| Past, < 5/day | 5 | 5 | 9 |
| Past, 5-10/day | - | 2 | 3 |
| Past, 11-20/day | - | - | 1 |
| Past, 21-30/day | - | - | - |
| Past, > 30/day | - | - | - |
| Present, < 5/day | 5 | 6 | 19 |
| Present, 5-10/day | 2 | - | 9 |
| Present, 11-20/day | - | - | 2 |
| Present, 21-30/day | - | - | - |
| Present, > 30/day | - | - | - |
| **BMI**, *n* |  |  |  |
| Very severely underweight (<14.9) | - | - | 2 |
| Severely underweight (15–14.9) | - | - | 2 |
| Underweight (16–18.5) | 7 | 4 | 12 |
| Normal (18.5–24.9) | 38 | 26 | 53 |
| Overweight (25–29.9) | 3 | 6 | 19 |
| Obese (≥30) | - | 2 | 7 |

*Note.* Medication status was categorized in terms of a single type of medication taken (anti-psychotic, anti-depressant, beta-blocker), as well as a separate level for any combination of various medication (13 levels in total). Smoking was coded in terms of a present or past smoking habit, as well as the number of cigarettes smoked (11 levels in total). Body-mass index (BMI) was classified in accordance with the World Health Organization criteria [42]. Abbreviations: CON = healthy controls; CHR-N = clinical high risk-negative; CHR-P = clinical high risk-positive.

**Supplementary Table 2.** Clinical sample characteristics.

|  | **CON** | **CHR-N** | **CHR-P** | **GROUP effect** | | | | **Pairwise comparisons** |
| --- | --- | --- | --- | --- | --- | --- | --- | --- |
|  | (n = 49) | (n = 38) | (n = 117) | F, *p* | η^2^ | | psihat / *p* (*p* crit) | |
| **CAARMS severity**, mean (SE) |  |  |  |  |  | | |  |
| Unusual Thought Content | - | 0.59 (0.18) | 1.77 (0.18) | 49.7, < **0.001***** | 0.20 | CHR-N vs CHR-P: -1.20 / < **0.001*****  CON vs CHR-P: -1.36 / < **0.001***** | | |
| Non-bizarre Ideas | - | 0.82 (0.17) | 2.85 (0.17) | 122.17, < **0.001***** | 0.42 | CHR-N vs CON: 0.64 / **0.002** (0.050)**  CHR-N vs CHR-P: -2.43 / < **0.001*****  CON vs CHR-P: -3.07 / < **0.001***** | | |
| Perceptual Abnormalities | - | 0.95 (0.21) | 2.81 (0.15) | 124.85, < **0.001***** | 0.43 | CHR-N vs CON: 0.56 / **0.006** (0.050)**  CHR-N vs CHR-P: -2.58 / < **0.001*****  CON vs CHR-P: -3.14 / < **0.001***** | | |
| Disorganized Speech | - | 0.54 (0.14) | 1.27 (0.13) | 53.42, < **0.001***** | 0.19 | CHR-N vs CHR-P: -0.82 / **0.005** (0.025)**  CON vs CHR-P: -1.06 / < **0.001***** | | |
| **CAARMS distress,** mean (SE) |  |  |  |  |  |  | | |
| Unusual Thought Content | - | 4.74 (2.22) | 23.84 (3.15) | nan | nan | CHR-N vs CHR-P: -12.93 / < **0.001*****  CON vs CHR-P:-12.93 / < **0.001***** | | |
| Non-bizarre Ideas | - | 15.18 (4.52) | 46.98 (3.58) | 53.33, < **0.001***** | 0.27 | CHR-N vs CHR-P: -42.52 / < **0.001*****  CON vs CHR-P:-47.00/ < **0.001***** | | |
| Perceptual Abnormalities | - | 8.08 (3.09) | 31.64 (3.36) | 38.67, < **0.001***** | 0.19 | CHR-N vs CHR-P: -23.26 / < **0.001*****  CON vs CHR-P:-23.86/ < **0.001***** | | |
| Disorganized Speech | - | 6.33 (2.49) | 18.40 (2.91) | nan | nan | CON vs CHR-P:-5.14 / **0.003** (0.016)****** | | |
| **GAF**, mean (SE) | 87.65 (0.93) | 69.87 (2.02) | 58.24 (1.23) | 185.45, < **0.001***** | 0.51 | CHR-N vs CON: -16.72 / < **0.001*****  CHR-N vs CHR-P: 13.62 / < **0.001*****  CON vs CHR-P: 30.34 / < **0.001***** | | |
| **BACS**, mean (SE) |  |  |  |  |  |  | | |
| Verbal memory | 52 (1.2) | 0.01 (0.2) | -0.35 (0.1) | 2.24, 0.112 | 0.02 |  | | |
| Digit sequencing | 21 (0.4) | 0.14 (0.2) | -0.13 (0.1) | 0.65, 0.523 | 0.00 |  | | |
| Token motor | 81 (1.7) | -0.66 (0.2) | -0.98 (0.1) | 13.58, **< 0.001***** | 0.10 | CHR-N vs CON: -0.62 / **0.012** (0.025)*  CON vs CHR-P: 0.95 / **< 0.001***** | | |
| Verbal fluency | 59 (2.0) | -0.22 (0.2) | -0.01 (0.1) | 0.68, 0.509 | 0.01 |  | | |
| Symbol coding | 74 (1.7) | 0.00 (0.2) | -0.58 (0.1) | 6.61, **0.002**** | 0.06 | CHR-N vs CHR-P: 0.58 / **0.005** (0.025)**  CON vs CHR-P: 0.67 / **< 0.001***** | | |
| Tower of London | 19 (0.2) | 0.15 (0.2) | -0.24 (0.1) | 1.40, 0.251 | 0.01 |  | | |
| Composite score | 304 (3.5) | -0.15 (0.2) | -0.61 (0.1) | 5.43, **0.006**** | 0.05 | CON vs CHR-P: 0.59 / **0.003** (0.017)** | | |
| **Emotion rec. test**, mean (SE) |  |  |  |  |  |  | | |
| Correct Response | 34.30 (0.4) | -0.19 (0.1) | -0.08 (0.1) | 0.46, 0.638 | 0.00 |  | | |
| Correct anger | 5.49 (0.2) | -0.00 (0.1) | -0.02 (0.1) | 0.02, 0.980 | 0.00 |  | | |
| Correct fear | 7.29 (0.2) | -0.19 (0.2) | -0.08 (0.1) | 0.45, 0.636 | 0.00 |  | | |
| Correct happy | 7.90 (0.1) | -0.19 (0.3) | -0.50 (0.2) | 2.10, 0.065 | 0.02 |  | | |
| Correct no emotion | 7.22 (0.2) | -0.23 (0.2) | -0.12 (0.1) | 0.52, 0.596 | 0.01 |  | | |
| Correct sad | 6.41 (0.1) | -0.01 (0.1) | 0.09 (0.1) | 0.28, 0.757 | 0.00 |  | | |
| Composite score | 68.61 (0.1) | -0.26 (0.2) | -0.21 (0.1) | 0.89, 0.414 | 0.01 |  | | |
| **CHR categories** |  |  |  |  |  |  | | |
| SPI-A BS criteria only | - | - | 27 |  |  |  | | |
| CAARMS UHR criteria only | - | - | 36 |  |  |  | | |
| CAARMS + SPI-A criteria | - | - | 49 |  |  |  | | |

*Note.* Welch’s ANOVA for unequal variance (F, alpha=0.05, 2-sided) and Hochberg-corrected, 20% means-trimmed, 3000 samples bootstrapped pairwise comparisons are reported [psihat / *p* (*p* crit)]. Eta-squared is reported as a measure of effect size. BACS and Emotion recognition test scores for clinical groups were standardized to control group data, controlled for sex category [45]. Abbreviations: CON = healthy controls; CHR-N = clinical high risk-negative; CHR-P = clinical high risk-positive; SE = standard error of mean.

**Supplementary Table 3.** Correlations of psychophysiological measurements with BS severity and distress.

|  | **BS Severity** | | | |  | **BS Distress** | | | |
| --- | --- | --- | --- | --- | --- | --- | --- | --- | --- |
|  | | **CON** | **CHR-N** | **CHR-P** |  | | **CON** | **CHR-N** | **CHR-P** |
| **RHR** | | - | 0.10  [-0.25, 0.44]  0.565 | 0.04  [-0.14, 0.22]  0.667 |  | | - | 0.19  [-0.11, 0.47]  0.260 | 0.07  [-0.12, 0.25]  0.467 |
| **RMSSD** | | - | 0.13  [-0.24, 0.44]  0.453 | -0.11  [-0.27, 0.07]  0.226 |  | | - | 0.01  [-0.32, 0.36]  0.949 | -0.10  [-0.29, -0.09]  0.296 |
| **SDNN** | | - | 0.14  [-0.20, 0.45]  0.397 | -0.11  [-0.29, 0.08]  0.229 |  | | - | 0.01  [-0.33, 0.35]  0.950 | -0.08  [-0.27, -0.11]  0.365 |
| **LF/HF** | | - | -0.19  [-0.48, 0.16]  0.250 | 0.05  [-0.14, 0.21]  0.611 |  | | - | -0.01  [-0.36, 0.35]  0.937 | -0.04  [-0.22, 0.14]  0.700 |

*Note.* Spearmans’s two-sided correlation with bias-corrected and accelerated (BCa) 2000 samples bootstrap 0.95 confidence interval, corrected for ties (ρ [95% CI], *p*). Abbreviations: CON = healthy controls; CHR-N = clinical high risk-negative; CHR-P = clinical high risk-positive; HRV = heart rate variability; SDNN = standard deviation of normal-to-normal heart beat intervals; RMSSD = square root of the mean squared differences of successive normal-to-normal intervals; HF = high frequency power.
